# Clinical Significance of Serum Galactose-Deficient IgA1 Level in Children with IgA Nephropathy

**DOI:** 10.1155/2020/4284379

**Published:** 2020-05-21

**Authors:** Hitoshi Irabu, Masaki Shimizu, Shuya Kaneko, Natsumi Inoue, Mao Mizuta, Kazuhide Ohta, Akihiro Yachie

**Affiliations:** ^1^Department of Pediatrics, Graduate School of Medical Sciences, Kanazawa University, Kanazawa, Japan; ^2^Department of Pediatrics, Kanazawa Medical Center, Kanazawa, Japan

## Abstract

This study was aimed at investigating the clinical significance of serum galactose-deficient IgA1 (Gd-IgA1) levels measured by a novel lectin-independent enzyme-linked immunosorbent assay (ELISA) using an anti-Gd-IgA1 monoclonal antibody (KM55) as a disease-specific biomarker for IgA nephropathy (IgAN) in children. Thirty-three children with IgAN, 40 with non-IgA glomerular diseases, and 38 age-matched healthy controls (HCs) were enrolled. Serum Gd-IgA1 levels were quantified by ELISA using KM55. Results were statistically compared with clinical features and pathological findings of IgAN. Serum Gd-IgA1 levels were significantly elevated in children with IgAN compared with children with non-IgA glomerular diseases and HCs. Serum Gd-IgA1 levels in children with IgAN were positively correlated with serum total IgA levels. However, the serum Gd-IgA1/total IgA ratio (Gd-IgA1/IgA) was also significantly elevated in children with IgAN. Serum Gd-IgA1 levels in children with IgAN increased in an age-dependent manner. The cutoff value of serum Gd-IgA1 levels for differentiating IgAN from non-IgA glomerular diseases was 3236 in children < 12 years and 5284 in children ≥ 12 years, respectively. In contrast, serum Gd-IgA1/IgA was age-independent. The cutoff value of serum Gd-IgA1/IgA for differentiating IgAN from non-IgA glomerular diseases was 0.2401. Serum Gd-IgA1 levels were negatively correlated with eGFR and positively correlated with mesangial IgA deposition. In contrast, serum Gd-IgA1/IgA levels were not correlated with any clinical parameters of IgAN. In conclusion, serum Gd-IgA1 levels were significantly elevated in children with IgAN. However, those levels were age-dependent; therefore, serum Gd-IgA1 levels classified by age and/or serum Gd-IgA1/IgA might have diagnostic values in children with IgAN.

## 1. Introduction

IgA nephropathy (IgAN) is the most common form of glomerular disease worldwide in children [1]. The main histopathological lesion characteristic of IgAN is IgA-dominant immunoglobulin deposits, which are often localized in the renal mesangial area [1]. These deposits are composed only of IgA1 subclass [2, 3]. The pathogenesis of IgAN is closely associated with aberrantly glycosylated IgA1 [4]. The IgA1 molecule has a hinge region, with nine potential O-glycosylation sites. O-glycosylation is required for the appropriate function of the IgA antibody [5]. O-glycosylation of IgA1 requires the addition of N-acetylgalactosamine (GalNAc) to serine or threonine residues of the IgA1 hinge region, followed by the addition of galactose [5]. Moreover, O-glycosylation is completed by the addition of sialic acid residues [5]. Although the underlying process has not been completely understood, impaired modifications of the IgA1 chain due to the abnormal expression or activity of glycosyltransferase make the O-glycosylated part of the IgA1 heavy-chain hinge region lack galactose and expose GalNAc residues [4]. Consequently, galactose-deficient IgA1 (Gd-IgA1) are formed. Gd-IgA1 tends to form polymeric structures [4]. Gd-IgA1 is also recognized by anti-Gd-IgA1 autoantibodies. This process results in the formation of circulating immune complexes. These complexes reduce liver clearance because of the large size of the complexes. Some complexes are deposited in the glomerular mesangium [5], subsequently activating mesangial cells to proliferate and overproduce extracellular matrix proteins and cytokines, thereby inciting injury of the glomerulus [6].

Previous studies have shown that serum Gd-IgA1 level is elevated in adults and pediatric patients with IgAN, demonstrating the severity of IgAN [7–11]. These findings suggest that the measurement of serum Gd-IgA1 level may be a helpful diagnostic test and could serve as a predictor of renal outcomes in IgAN. However, proving Gd-IgA1 as a biomarker has still remained controversial, according to a recent meta-analysis [12]. The reason for this controversy is the absence of a definitive assay for the measurement of Gd-IgA1. Previously, serum Gd-IgA1 had been conventionally quantified using a lectin-based enzyme-linked immunosorbent assay (ELISA) with GalNAc-specific lectin extracted from *Helix aspersa* (HAA) [7, 8] or *Vicia villosa* [9]. A lectin-based assay, in particular, the HAA lectin-based assay, has been a useful tool for clinical and basic research regarding the pathology, diagnosis, and treatment of IgAN for years. However, the HAA lectin-based assay has several limitations. One of these is that its bioactivity and stability depend on the product lot of lectin, because HAA lectins can be isolated from a natural source and supplied by manufacturers as a highly purified protein by affinity chromatography. Therefore, a more robust assay for detecting circulating Gd-IgA1 is desired. Recently, a novel lectin-independent ELISA was developed [13]. This ELISA makes use of an anti-Gd-IgA1 monoclonal antibody (KM55) that can be steadily obtained from hybridoma cells. However, a limited number of studies have utilized this new assay to measure serum Gd-IgA1 level in children with IgAN [10]. Therefore, whether serum might have diagnostic and prognostic values in children with IgAN is still unknown, as well as the cutoff value of serum Gd-IgA1 levels for differentiating IgAN from non-IgA glomerular diseases. In the present study, we evaluated serum Gd-IgA1 level using KM55 and attempted to clarify the clinical significance of Gd-IgA1 in children with IgAN including diagnostic and prognostic values.

## 2. Materials and Methods

### 2.1. Patients and Samples

We enrolled 73 children with primary/secondary glomerulopathy. Renal biopsy was used for the diagnosis of each disease other than minimal change (MC). The diagnosis of MC was based on good response for steroid in remission induction for nephrotic syndrome. Serum samples were obtained from 33 children with IgAN, 40 with non-IgA glomerular diseases including 20 with MC, 6 with focal segmental glomerulosclerosis (FSGS), 3 with membranous nephropathy (MN), 11 with lupus nephritis (LN), and 38 age-matched healthy controls (HCs). These samples from children with each disease other than MC were collected at the time of renal biopsy. Serum samples from children with MC were collected at the time of the acute phase of nephrotic syndrome. The clinical characteristics of the patients are summarized in Table 1.

4 *μ*m thick frozen kidney sections were fixed in cold acetone for the immunofluorescence study for mesangial IgA deposition. IgA deposition was classified into point 0 to point 3 as follows: point 0, no staining; point 1, weak; point 2, moderate; and point 3, strong. Four nephrologists examined the specimens independently and assessed the staining patterns.

The histological activity and chronicity scores of IgAN were extracted from a grading system of specific histological features [14]. The activity index was assessed as follows: (1) cellular proliferation was graded as 0–3, (2) necrosis as 0 or 1 (present or absent), (3) interstitial mononuclear cell infiltration as 0–3, and (4) cellular crescent formation as 0–3, according to the percentage of involved glomeruli with crescents (0%, 0; 1%–20%, 1; 20%–50%, 2; and >50%, 3). The chronicity index was assessed as follows: fibrous crescent formation and segmental and global sclerosis were scored as 0–3, according to the percentage of involved glomeruli with crescents (0%, 0; 1%–20%, 1; 20%–50%, 2; and >50%), and atrophic tubuli and/or interstitial fibrosis were graded as 0–3 (0%, 0; 1%–20%, 1; 20%–50%, 2; and >50%). The sum of these scores comprised the activity and chronicity index (maximum 10).

This study was approved by the Institutional Review Board of Kanazawa University, and informed consent was obtained from the participants and the guardians of the children.

### 2.2. Enzyme-Linked Immunosorbent Assay for Detecting Serum Gd-IgA1 Level

Serum Gd-IgA1 level was assessed by ELISA according to the manufacturer's instructions (#27600; Immuno-Biological Laboratories, Fujioka, Japan).

### 2.3. Urinalysis

Hematuria was assessed by the number of red blood cells per high power field in a fresh urine sample. Significant hematuria was defined as ≥5 red blood cells per high power field. Proteinuria was quantified using the urinary protein/creatinine ratio of the first morning urine sample. Significant proteinuria was defined as urinary protein/creatinine ratio ≥ 0.2.

### 2.4. Statistical Analysis

Data are expressed as medians. Serum Gd-IgA1 levels were skewed. Hence, these values were normalized by log transformation before interpretation. Histograms of original and log-transformed data of serum Gd-IgA1 levels are shown in the Supplement Figure (available here). Comparisons among the groups were performed using one-way analysis of variance with Tukey's multiple comparison test. Furthermore, correlations were expressed using Spearman's rank correlation coefficient. A *p* value of <0.05 was considered statistically significant.

## 3. Results

### 3.1. Increased Serum Gd-IgA1 Level in Children with IgAN

**Figure 1:**
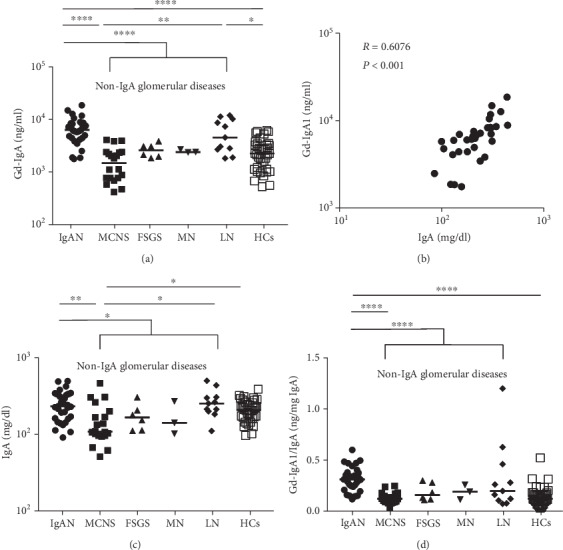
Serum Gd-IgA1 levels, serum total IgA levels, and serum Gd-IgA1/total IgA ratio in children with various glomerulopathies. (a) Serum Gd-IgA1 levels. (b) The correlation between serum Gd-IgA1 levels and serum total IgA levels. (c) Serum total IgA levels. (d) Serum Gd-IgA1/total IgA ratio. Bars represent median values. Statistically significant differences between each patient group are shown as ^∗^*p* < 0.05, ^∗∗^*p* < 0.01, and ^∗∗∗∗^*p* < 0.0001. IgAN: IgA nephropathy; MC: minimal change; FSGS: focal segmental glomerulosclerosis; MN: membranous nephropathy; LN: lupus nephritis; HCs: healthy controls.

As shown in Figure 1(a), serum Gd-IgA1 levels were significantly elevated in patients with IgAN (median, 6310 ng/ml; range, 1738–18621 ng/ml) compared to children with non-IgA glomerular diseases (2344 ng/ml, 407–12022 ng/ml) (*p* < 0.0001) and HCs (2239 ng/ml, 513–6166 ng/ml) (*p* < 0.0001). The levels in children with MC (1413 ng/ml, 407–4074 ng/ml), FSGS (2512 ng/ml, 1820–3802 ng/ml), and MN (2398 ng/ml, 2344–2692 ng/ml) were not remarkably increased compared with those in HCs. Serum Gd-IgA1 levels were significantly elevated in patients with LN (4570 ng/ml, 1820–12022 ng/ml) compared with patients with MC (*p* < 0.01) and HCs (*p* < 0.05).

As shown in Figure 1(b), serum Gd-IgA1 levels were positively correlated with serum total IgA levels in patients with IgAN (*p* < 0.001, *R* = 0.6076). As shown in Figure 1(c), serum total IgA levels were significantly elevated in children with IgAN (215 mg/dl, 86-450 mg/dl) compared with those with non-IgA glomerular diseases (156 mg/dl, 49-455 mg/dl) and those with MC, although those levels were not elevated compared to those in HCs (193 mg/dl, 91-355 mg/dl). Serum total IgA levels in each non-IgA glomerular disease were as follows: MC (102 mg/dl, 49-423 mg/dl), FSGS (155 mg/dl, 105-280 mg/dl), MN (132 mg/dl, 96-249 mg/dl), and LN (233 mg/dl, 104-455 mg/dl).

Next, we adjusted serum Gd-IgA1 levels for total IgA levels. As shown in Figure 1(d), serum Gd-IgA1 levels/total IgA levels (Gd-IgA1/IgA) were significantly elevated in children with IgAN (0.297 ng/mg IgA, 0.110-0.575) compared to those with non-IgA glomerular diseases (0.127, 0.027-1.155) (*p* < 0.0001) and HCs (0.115, 0.025-0.500) (*p* < 0.0001). Serum Gd-IgA1/IgA in each non-IgA glomerular disease were as follows: MC (0.114, 0.027-0.235), FSGS (0.150, 0.103-0.287), MN (0.181, 0.107-0.245), and LN (0.188, 0.067-1.155).

### 3.2. Age-Dependent Increase in Serum Gd-IgA1 Levels in Children with IgAN

**Figure 2:**
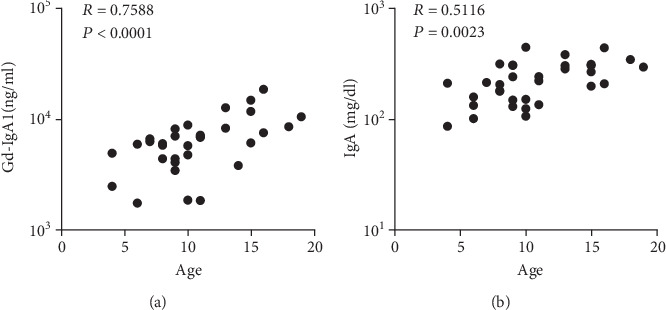
Correlation between age and serum Gd-IgA1 levels and serum total IgA levels in children with IgA nephropathy: (a) serum Gd-IgA1 levels; (b) serum total IgA levels.

Serum Gd-IgA1 levels in children with IgAN were positively correlated with age (*p* < 0.0001, *R* = 0.7588) (Figure 2(a)). Furthermore, serum total IgA levels in children with IgAN were positively correlated with age (*p* < 0.01, *R* = 0.5116) (Figure 2(b)).

**Figure 3:**
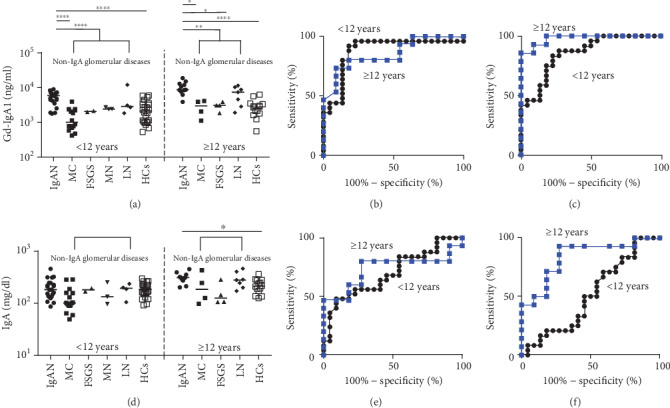
Serum Gd-IgA1 levels and serum total IgA levels classified by age. (a) Serum Gd-IgA1 levels classified by age. (b) The receiver operating characteristic curve analysis of serum Gd-IgA1 levels for differentiating IgA nephropathy from other glomerular diseases. (c) The receiver operating characteristic curve analysis of serum Gd-IgA1 levels for differentiating IgA nephropathy from healthy controls. (d) Serum IgA levels classified by age. (e) The receiver operating characteristic curve analysis of serum IgA levels for differentiating IgA nephropathy from other chronic kidney diseases. (f) The receiver operating characteristic curve analysis of serum IgA levels for differentiating IgA nephropathy from healthy controls. Bars represent median values. Statistically significant differences between each patient group are shown as ^∗^*p* < 0.05, ^∗∗∗^*p* < 0.001, and ^∗∗∗∗^*p* < 0.0001. IgAN: IgA nephropathy; MC: minimal change; FSGS: focal segmental glomerulosclerosis; MN: membranous nephropathy; LN: lupus nephritis; HCs: healthy controls; CKD: chronic kidney diseases.

Serum Gd-IgA1 levels in children with IgAN increased in an age-dependent manner (Figure 3(a)). Serum Gd-IgA1 levels in children with IgAN < 12 years were significantly elevated (5888 ng/ml, 1738–8913 ng/ml) compared with those in children with non‐IgA glomerular diseases < 12 years (1995 ng/ml, 407–12022 ng/ml) (*p* < 0.0001) and HCs < 12 years (513 ng/ml, 2042–5888 ng/ml) (*p* < 0.001) as well as those in children with IgAN ≥ 12 years (8709 ng/ml, 3802–18621 ng/ml) compared with children with non‐IgA glomerular diseases ≥ 12 years(3802 ng/ml, 1096–11482 ng/ml) (*p* < 0.05) and HCs ≥ 12 years (2692 ng/ml, 537–6166 ng/ml) (*p* < 0.001). Serum Gd-IgA1 levels in each non-IgA glomerular disease were as follows: for patients < 12 years, MC (977 ng/ml, 407–3890 ng/ml), FSGS (2042 ng/ml, 1950–2089 ng/ml), MN (2399 ng/ml, 2344–2692 ng/ml), and LN (2818 ng/ml, 1820–12022 ng/ml) and for patients ≥ 12 years, MC (2818 ng/ml, 1096–4074 ng/ml), FSGS (3090 ng/ml, 1820–3802 ng/ml), and LN (7413 ng/ml, 1862–11482 ng/ml).

The receiver operating characteristic curve analysis indicated that the cutoff for serum Gd-IgA1 levels for differentiating IgAN from non-IgA glomerular diseases was 3236 ng/ml with a sensitivity of 92% and a specificity of 81.8% in children < 12 years and 5284 ng/ml with a sensitivity of 73.3% and a specificity of 90.9% in children ≥ 12 years, respectively (Figure 3(b)). The area under the ROC curve and 95% confidence interval were as follows: 0.8764 and 0.766–0.9867 (<12 years) and 0.8515 and 0.7049–0.9981 (≥12 years). The cutoff for serum Gd-IgA1 levels for differentiating IgAN from HCs was 3350 ng/ml with a sensitivity of 75% and a specificity of 81.8% in children < 12 years and 5861 ng/ml with a sensitivity of 92.9% and a specificity of 90.9% in children ≥ 12 years, respectively (Figure 3(c)). The area under the ROC curve and 95% confidence interval were as follows: 0.858 and 0.7523-0.9636 (<12 years) and 0.9805 and 0.9387–1.022 (≥12 years).

Serum total IgA levels in children with IgAN also increased in an age-dependent manner (Figure 3(d)). Serum total IgA levels in children with IgAN < 12 years were not significantly elevated (180 mg/dl, 86-450 mg/dl) compared with those in children with non‐IgA glomerular diseases < 12 years (129 mg/dl, 49-281 mg/dl) and HCs < 12 years (183 mg/dl, 91-295 mg/dl). In contrast, serum total IgA levels in children with IgAN ≥ 12 years (306 mg/dl, 199-444 mg/dl) were significantly elevated compared with those in HCs ≥ 12 years (210 mg/dl, 124-355 mg/dl) (*p* < 0.05), although those were not significantly elevated compared with those in patients with CKD ≥ 12 years (241 mg/dl, 97-455 mg/dl). Serum total IgA levels in each non-IgA glomerular disease were as follows: for patients < 12 years, MC (101 mg/dl, 49-423 mg/dl), FSGS (179 mg/dl, 167-191 mg/dl), MN (132 mg/dl, 96-249 mg/dl), and LN (192 mg/dl, 104-233 mg/dl) and for patients ≥ 12 years, MC (183 mg/dl, 97-423 mg/dl), FSGS (125 mg/dl, 105-280 mg/dl), and LN (275 mg/dl, 170-455 mg/dl).

The receiver operating characteristic curve analysis indicated that the cutoff for serum total IgA levels for differentiating IgAN from non-IgA glomerular diseases was 158 ng/ml with a sensitivity of 64.0% and a specificity of 59.1% in children < 12 years and 283 ng/ml with a sensitivity of 80.0% and a specificity of 72.7% in children ≥ 12 years, respectively (Figure 3(e)). The area under the ROC curve and 95% confidence interval were as follows: 0.6973 and 0.5471–0.8475 (<12 years) and 0.7333 and 0.5321–0.9346 (≥12 years).

The cutoff for serum total IgA levels for differentiating IgAN from HCs was 206 ng/ml with a sensitivity of 62.5% and a specificity of 45.5% in children < 12 years and 286 ng/ml with a sensitivity of 92.9% and a specificity of 72.7% in children ≥ 12 years, respectively (Figure 3(f)). The area under the ROC curve and 95% confidence interval were as follows: 0.5114 and 0.3386-0.6842 (<12 years) and 0.8377 and 0.6752–1.0000 (≥12 years).

### 3.3. Serum Gd-IgA1/Serum Total IgA Ratio as an Age-Independent Marker for the Diagnosis of IgAN

**Figure 4:**
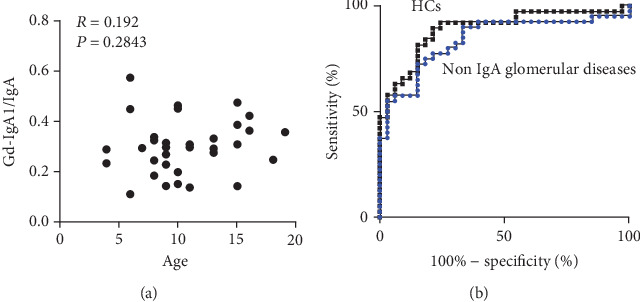
Serum Gd-IgA1/total IgA ratio as an age-independent biomarker for the differentiation between IgA nephropathy and non-IgA glomerular disease. (a) Correlation between age and serum Gd-IgA1/total IgA ratio. (b) The receiver operating characteristic curve analysis of the serum Gd-IgA1/total IgA ratio for differentiating IgA nephropathy from other non-IgA glomerular diseases and healthy controls.

As shown in Figure 4(a), Gd-IgA1/IgA was not affected by age. The receiver operating characteristic curve analysis indicated that the cutoff for Gd-IgA1/IgA for differentiating IgAN from non-IgA glomerular diseases was 0.2401 with a sensitivity of 80% and a specificity of 72.73% (Figure 4(b)). The area under the ROC curve and 95% confidence interval were 0.8402 and 0.746–0.9343, respectively. The cutoff for Gd-IgA1/IgA for differentiating IgAN from HCs was 0.1782 with a sensitivity of 81.6% and a specificity of 84.9% (Figure 4(b)). The area under the ROC curve and 95% confidence interval were 0.8915 and 0.8149–0.9682, respectively.

### 3.4. Relationship between Serum Gd-IgA1 Level and Clinical Parameters of IgAN

**Figure 5:**
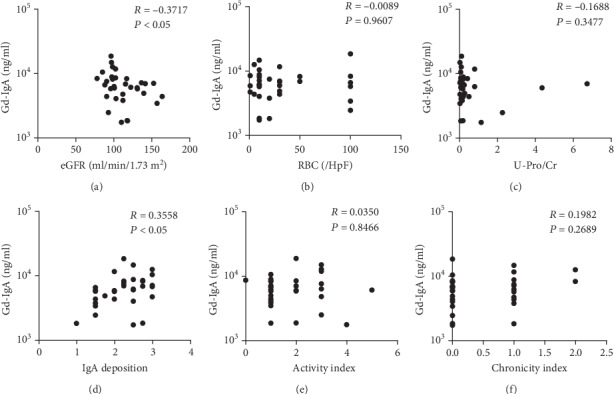
Correlation between serum Gd-IgA1 levels and clinical and histological parameters in patients with IgA nephropathy: (a) eGFR, (b) hematuria, (c) proteinuria, (d) mesangial IgA deposition, (e) activity index, and (f) chronicity index. RBC: red blood cells; HpF: high-power field; U-Pro/Cr: urinary protein to creatinine ratio.

Serum Gd-IgA1 levels were negatively correlated with eGFR (*p* < 0.05, *R* value = −0.3633) (Figure 5(a)). In contrast, serum Gd-IgA1 levels were not correlated with the degree of hematuria (Figure 5(b)) and proteinuria (Figure 5(c)). Serum Gd-IgA1 levels were positively correlated with the degree of mesangial IgA deposition (Figure 5(d)). However, serum Gd-IgA1 levels were not correlated with the histological severity of IgAN including mesangial proliferation, crescent formation, glomerular sclerosis, tubular atrophy, interstitial fibrosis, and activity index (Figure 5(e)) and chronicity index (Figure 5(f)).

**Figure 6:**
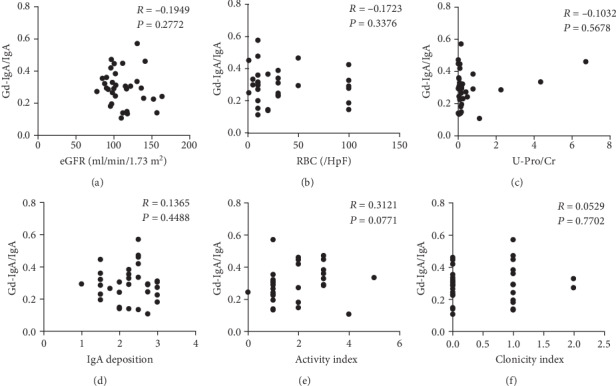
Correlation between the serum Gd-IgA1/total IgA ratio and clinical and histological parameters in patients with IgA nephropathy: (a) eGFR, (b) hematuria, (c) proteinuria, (d) mesangial IgA deposition, (e) activity index, and (f) chronicity index. RBC: red blood cells; HpF: high-power field: U-Pro/Cr: urinary protein to creatinine ratio.

Serum Gd-IgA1/IgA levels were not correlated with clinical and histological parameters including eGFR (Figure 6(a)) and the degree of hematuria (Figure 6(b)) and proteinuria (Figure 6(c)). Serum Gd-IgA1 levels were not correlated with the degree of mesangial IgA deposition (Figure 6(d)) and the histological severity of IgAN including mesangial proliferation, crescent formation, glomerular sclerosis, tubular atrophy, interstitial fibrosis, and activity index (Figure 6(e)) and chronicity index (Figure 6(f)).

## 4. Discussion

Renal biopsy is essential for diagnosing IgAN and assessing the degree of inflammation and tubulointerstitial damage. However, biopsy is invasive, requires hospitalization, and is rarely associated with serious complications. Therefore, a noninvasive diagnostic approach that compensates for the disadvantages of biopsy is desired for patients with IgAN, particularly for children.

Based on multihit pathogenesis of IgAN, circulating Gd-IgA1 and IgA1 containing ICs are critical for mesangial IgA1 deposition [4]. Although the exact mechanism is still unknown, altered and sustained C1GALT1 and ST6GALNAC2 enzyme activities due to genetic or environmental factors might be closely associated with Gd-IgA1 overproduction in IgAN, leading to an increase in circulating Gd-IgA1 and mesangial IgA deposition in patients with IgAN [4].

Previous studies using conventional lectin assays showed that serum Gd-IgA1 levels were significantly elevated in children with IgAN and serum Gd-IgA1 levels can differentiate IgAN from other kidney diseases and could also serve as a powerful diagnostic approach for diagnosing IgAN [7–9]. In this study, we measured serum Gd-IgA1 levels using a novel lectin-independent ELISA making use of an anti-Gd-IgA1 monoclonal antibody (KM55). We showed that serum Gd-IgA1 levels were significantly elevated in children with IgAN. The results in this study are consistent with previous studies using KM55 [10] as well as studies using conventional lectin assays [7–9]. The ELISA using KM55 has some advantages compared with conventional lectin assays. KM55 can be steadily obtained from hybridoma cells. Therefore, this assay is more robust for detecting circulating Gd-IgA1, because lectins are isolated from a natural source and bioactivity and stability depend on the product lot of lectin. Furthermore, it takes only 2 hours to measure serum Gd-IgA1 levels in the ELISA using KM55, whereas lectin-based assays need much longer time. From these results, the ELISA using KM55 has a great diagnostic utility for IgAN.

In this study, serum Gd-IgA1 levels were elevated not only in children with IgAN but also in children with LN. These findings were observed in a previous study using KM55, although serum Gd-IgA1 levels were significantly elevated in patients with IgAN compared to patients with LN [11]. Therefore, careful interpretation of serum Gd-IgA1 levels is of great importance in LN patients with atypical clinical manifestations.

In this study, serum Gd-IgA1 levels increased in an age-dependent manner and were positively correlated with age. Therefore, serum Gd-IgA levels should be evaluated with the cutoff values classified by age. The receiver operating characteristic curve analysis indicated that the cutoff for serum Gd-IgA1 levels for differentiating IgAN from non-IgA glomerular diseases was 3236 ng/ml with a sensitivity of 92% and a specificity of 81.8% in children < 12 years and 5284 ng/ml with a sensitivity of 73.3% and a specificity of 90.9% in children ≥ 12 years, respectively. Furthermore, serum Gd-IgA levels had a higher area under the ROC curve value compared with serum total IgA levels. Therefore, serum Gd-IgA levels might be a more accurate biomarker for the diagnosis of IgA compared to serum total IgA levels. From these findings, serum Gd-IgA1 level > 3236 ng/ml in children < 12 years and 5284 ng/ml in children ≥ 12 years might have diagnostic and prognostic values in children with IgAN.

In contrast, Gd-IgA1/IgA was not affected by age. The receiver operating characteristic curve analysis indicated that the cutoff for Gd-IgA1/IgA for differentiating IgAN from non-IgA glomerular diseases was 0.2401 with a sensitivity of 80% and a specificity of 72.73%. Although the value of the area under the ROC curve of Gd-IgA1/IgA was lower compared with that of serum Gd-IgA levels, Gd-IgA1/IgA was also an age-independent useful biomarker for differentiating IgAN from non-IgA glomerular diseases.

In the present study, serum Gd-IgA1 levels were not correlated with the degree of proteinuria. This result is consistent with those in several previous studies [8–11]. Conversely, other reports concentrating on adults showed that serum Gd-IgA1 levels are significantly associated with the degree of proteinuria [15, 16]. A recent study reported that urinary Gd-IgA1 level is positively correlated with the degree of proteinuria [17]. Furthermore, urinary IgA1 rather than serum IgA1 level is associated with a higher degree of galactose deficiency [17]. Although we did not assess urinary Gd-IgA1 level, these findings indicate that urinary Gd-IgA1 level might be more sensitive for evaluating the severity of IgAN. In addition, several studies have revealed that serum Gd-IgA1-specific antibodies, rather than Gd-IgA1 itself, and Gd-IgA1 containing ICs were significantly elevated in patients with a high number of crescents or the degree of mesangial IgA deposition [18]. Further studies are warranted to clarify the clinical significance of the level of urinary Gd-IgA1 or serum-specific antibodies to Gd-IgA1 as a promising biomarker for the severity of IgAN.

In this study, serum Gd-IgA1 levels were negatively correlated with eGFR. This result was consistent with previous studies focused on adults and children [10, 11]. However, Gd-IgA1/IgA was not correlated with eGFR. This result was consistent with a recent systematic review which showed that there is no correlation between serum Gd-IgA1 level and renal dysfunction [12]. Although the reason for this discrepancy is unclear, in this study, most children with IgAN showed normal or almost normal renal function. Further long-term observation studies are needed to clarify the prognostic value of serum Gd-IgA levels for IgAN.

The present study has several limitations. Firstly, we collected data only at the time of biopsy. Secondly, this is a single-center study involving only Japanese patients. Recent studies to identify genetic factors contributing to levels of Gd-IgA1 showed the pathogenic importance of changes in IgA1 O-glycosylation which may vary in different ethnicities [19, 20]. KM55 recognizes a specific epitope in the hinge region of the IgA1 molecule. Therefore, the data in this study might not necessarily be extrapolated to other ethnic groups. Thirdly, the present study included a small number of patients and HCs. This study was an exploratory, retrospective study and lacking independent validation. A study with a larger sample size including different ethnicities may help to define the true value of serum Gd-IgA1 level as a diagnostic biomarker for IgAN.

In conclusion, serum Gd-IgA1 levels were significantly elevated in children with IgAN. However, those levels were age-dependent; therefore, serum Gd-IgA1 levels classified by age and/or serum Gd-IgA1/total IgA ratio might have diagnostic values in children with IgAN.

## Figures and Tables

**Table 1:** Clinical characteristics of children with various glomerulopathies.

	IgAN	MC	FSGS	MN	LN	HCs
Number	33	20	6	3	11	38
Age (mean, years)	10.6 ± 3.9	6.6 ± 4.0	12.7 ± 3.3	7.7 ± 2.1	12.2 ± 3.1	9.8 ± 4.3
Sex (female/male)	17/16	8/12	5/1	1/2	11/0	16/22
Proteinuria, *n* (%)	16 (48.5)	20 (100)	5 (83.3)	3 (100)	9 (81.8)	0
Hematuria, *n* (%)	32 (97.0)	14 (70.0)	1 (16.7)	0 (0)	4 (36.3)	0
Elevated serum creatinine levels for age	0	0	0	0	0	0

## Data Availability

All data generated or analysed during this study are included in this published article.

## Abbreviations

Gd-IgA1: Galactose-deficient IgA1
ELISA: Enzyme-linked immunosorbent assay
IgAN: IgA nephropathy
CKD: Chronic kidney diseases
HCs: Healthy controls
MC: Minimal change
FSGS: Focal segmental glomerulosclerosis
MN: Membranous nephropathy
LN: Lupus nephritis.

## Supplementary Materials

Supplementary Materials Histograms of original and log-transformed data of serum Gd-IgA1 levels.
