# Supplementary figures and images for: Clinical Significance of Serum Galactose-Deficient IgA1 Level in Children with IgA Nephropathy
Source: J Immunol Res. 2020 May 21;2020:4284379. doi: 10.1155/2020/4284379 (PMC7260647; doi:10.1155/2020/4284379)

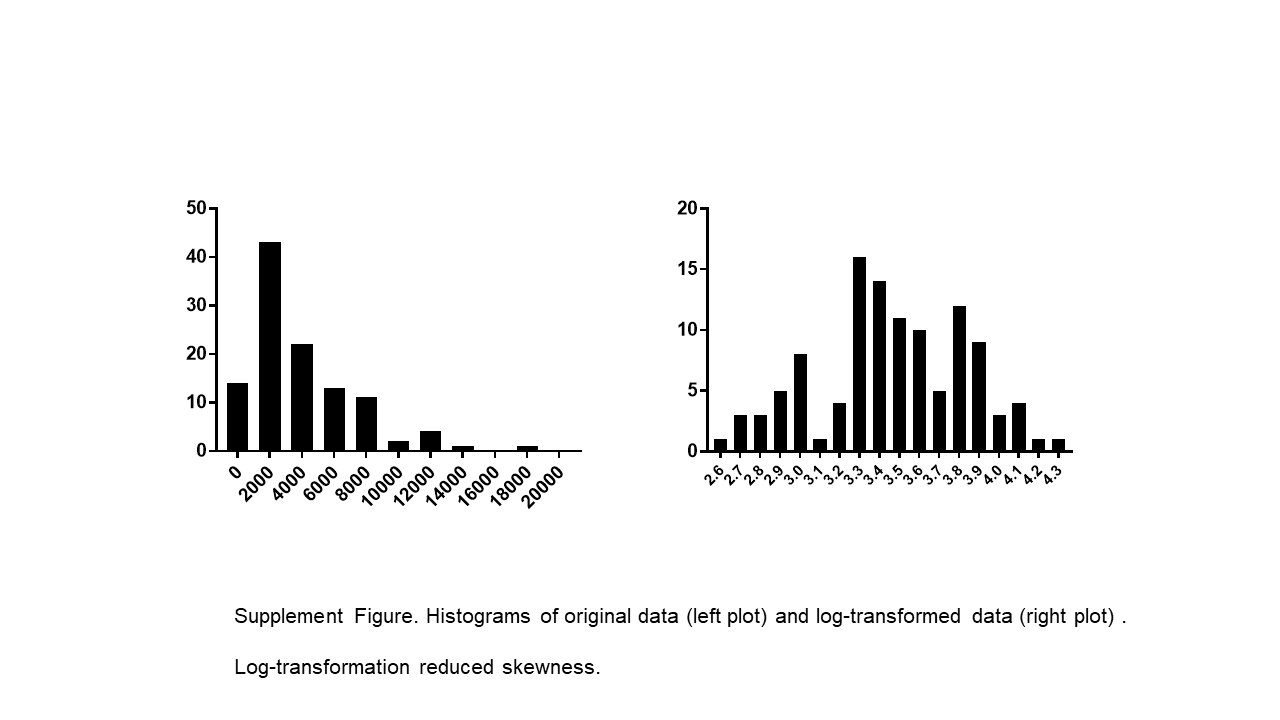

Supplement: Supplementary Materials — Histograms of original and log-transformed data of serum Gd-IgA1 levels. [file 4284379.f1.tif]
